# Galactagogue and Emmenagogue Effects of Warm and Stimulating Applications to the Mammæ

**Published:** 1853-09

**Authors:** 


*Galactagogue and Emmenagogue Effects of warm and stimulating Applications to the Mammæ.*—Dr. J. R. Cormack endeavors to establish (*Association Medical Journal,* March 25, 1853,) by the brief narrative of a few facts, the following propositions:—

1. Warmth and stimulants applied to the mammæ often act powerfully as *galactagogues.*

2. Warmth and stimulants applied to the mammæ often act powerfully as *emmenagogues.*

3. The leaves of the bofareira (or ricinus communis) and jatropha curcas act as galactagogues and emmenagogues; but not from their possessing any peculiar or specific power.

I. *Warmth and stimulants applied to the Mammæ often act powerfully as Galactagogues.* CASE I. *Milk restored to the Mammæ by hot Fomentations.*—Last month, a lady, when nursing her infant, about seven months old, was attacked with acute bronchitis of moderate severity, which was successfully treated by low diet, and tartar-emetic in small doses. At the end of four days, the bronchitis was cured; but the milk, which had previously been failing, almost entirely left the breasts. On the fifth day, from exposure to cold, she experienced a relapse of the bronchial affection. As she had been considerably weakened by the previous attack, and as the symptoms of the relapse were not sufficently severe to justify recourse a second time to antimony, I ordered her to take a draught containing ammonia and chloroform, as an anodyne, expectorant, and diaphoretic, every eight hours; and to carry out similar intentions, I also directed a succession of pillow-cases, filled with heated moist bran, to be applied to the chest. When I saw her on the following day, after this treatment had been employed, she told me that she had profusely perspired for some hours; she was (after copious expectoration) free from cough, and pain in the chest; and, what was equally a source of pleausure and surprise to her, *her breasts had become distended with milk* This lady was able to resume nursing, and to continue it with the assistance of a suitable diet.

CASE II. *Effects of a sinapism applied to one of the Mammæ of a lady five months advanced in Pregnancy: effects of warmth in the same case*—A lady was under my care for bronchitis, at the same time as the patient whose case I have just sketched. She was directed one night to apply a sinapism over the sternum, which she did; but having fallen asleep, it slipped to the side, and remained undisturbed for about an hour upon one of the breasts. For some days, this mamma was very much larger in size than the other, and its areola was also much darker. From the delicacy of this lady, and the unusual severity of the weather, I directed her to wear a double flannel jacket, and a wadded wrapper round the chest. She tells me that

*the breasts are larger, and the areolæ much deeper in color than they ever were in any of her ten previous pregnancies even at the full time*; and these conditions were established while her general health was exceedingly depressed by illness.

CASE III. *Stimulating Embrocation increasing the Supply of Milk.*—A lady, though in excellent health, had a very scanty supply of milk for her infant, when it was a few weeks old; She consulted me as to the use of means to remedy this evil; and I advised her to rub the mammæ gently, every six or eight hours, with an embrocation containing a small quantity of tincture of cantharides and oil of thyme, and to sheath the mammæ very warmly in wadding *In a few days the milk was abundant.*

CASE IV. *Hot poultices keeping up the Secretion of Milk when this was not desired.* A lady suffered, after her confinement, from a succession of abscesses and abortive abscesses in the breast. The surgeon who attended her treated her by antiphlogistic medicines, under which discipline she passed some wretched months, from mental and bodily depression, aggrvated by hysterical attacks. The local affection did not seem to make any satisfactory progress; and the great obstacle to a cure was stated to be the impossibility of getting rid of the milk, in spite of saline purges being freely administered. The mammæ during the whole of the period to which I refer, had been ceaselessly treated, night and day, with hot poultices and medicated fomentations. These applications were abandoned, and a generous diet prescribed. *In a few days there was not a drop of milk in the breasts*; and the abscesses, actual and threatening, had ceased to give any pain, and had, in fact, almost disappeared.

I might refer to other cases, which I have vividly in my memory; but the above, which have occured within the last three months, seem sufficient to establish the first proposition; viz., that *warmth and stimulants applied to the mammæ often act powerfully as galactagogues.* I need hardly add, that along with the use of such means, the regular application of an infant to the breast would greatly assist in reproducing lactation, as, according to the testimony of various authors, this stimulus has of itself proved sufficient to restore the secretion of milk, and has actually caused it to flow, not only from virgins and other women who had never been pregnant, but even from males.* Excitement and sanguineous turgescene of the gland is induced; and these conditions afford to the organ both a power and a stimulus to perform its previously dormant function.

II. *Warmth and Stimulants applied to the Mammæ, often act powerfully as Emmenagogues.* Warm clothing of the abdomen and limbs, hot hip-baths and medicnes which stimulate the badder and rectum (such as ergot, cantharides, and aloes), have undoubted emmenagogue powers in properly selected cases of retarded or suppressed catamenia; and, indeed, they constitute, in various combinations, the principle measures by which the physician usually endeavours to excite the ovarian nisus upon which menstruation depends. The observant physician knows well, that while his treatment is directed to the uterus through the ovaries, the effects produced upon the mammæ are generally very striking, and the first indications which he expects to find of the uterus being roused from its torpor are turgescence and tingling of the mammæ; phenomena which also usually precede normal menstruation. It is equally true, though not so familiarly understood, that measures which act directly and primarily upon the breasts, such as warm clothing to the bust, and the application of stimulants, not only cause them to swell and throb, but likewise stimulate the ovaries, and cause menses to flow. The practice adopted by some practitioners, of applying leeches to the mammæ in amenorrhœa, owes its effiacy to fomentations used, and the irritation of the bites.

In 1834, Dr. Charles Patterson published, in the *Dublin Journal of Medicine*, a paper in which he described the emmenagogue power of irritation of the mammæ by sinapisms. This paper fell into my hands at the time of its appearance, when I was an Edinburgh dispensary pupil, practising I believe, with more zeal than knowledge, and using, often with more confidence than discrimination, that plan of treatment which I had seen most recently or most enthusiastically recommended. In these circumstances, I successfully employed Dr. C. Patterson's method in

---

*Cases in which men have suckled children are on record. The essential character of the gland is the same in both sexes.

amenorrhœa. The beneficial results which I then obtained, produced a very strong impression upon my mind as to the effiacy of irritation of the mammæ in producing menstruation; and the experience of nineteen maturer years has confirmed this impression.

As Dr. Patterson's facts do not seem to be referred to by subsequent writers, and as the practice which he recommends is so little noticed by authors, I subjoin an extract from his paper. Dr. Patterson writes as follows:—

"Mary Reardon, aged 24, of moderately corpulent habits, was admitted into the Rathkeale Hospital on the 10th of August, 1832. She laboured under slight synochial fever, which in a few days yielded to venesection and purgatives. On the 19th of August, smymptoms which were considered of an hysterical character presented themselves, with pain in the upper and outer part of the right side of the chest. For the latter affection, a small sinapism was prescribed; but from inattention of the nurse, it was made so large that it covered a considerable portion of the mamma. The sinapism remained on for half an hour. At the visit on the following morning, the 20th August, Reardon complained that the right breast was exceedingly painful— the pain being very different in its character from that which she had before experienced. On examination, the whole side of the chest was found considerably swollen: there was slight diffused redness of the skin: and though the mamma itself was enlarged to four or five times its natural bulk, yet there was no circumscribed hardness, nor any tendency to suppurative inflammation. On the 21st August, the right mamma and adjoining parts of the chest were found much more enlarged than they had been at the preceding visit. The left mamma and side of the thorax were unaffected; and it was announced by the nurse that the catamenia had that morning appeared, and were then in considerable quantity. This discharge, which, as the patient stated, had been for two years and a half wholly suppressed, continued to flow for two days; then it began to decline, and with it the tumefaction of the mamma gradually disappeared."

Dr. Patterson's attention having thus accidentally been directed to mammary irritation as an excitant of the torpid uterus, he resolved to try its effiacy when a suitable opportunity presented itself. His next case is thus described:—

"Catherine Power, aged 19, applied to me on the 14th September, 1832. She complained of headache, languor, loss of appettie, and inability to attend to her usual business, that of a servant. She stated, that about the middle of April, the menstrual discharge being then present, she incautiously exposed herself to cold in washing clothes at the river. The catamenia then suddenly ceased, and had not since returned; and from that period she had been constantly subject to ill health. She had consulted different medical gentlemen, and taken a great variety of medicine, with little advantage. I directed that the clavicular half of the right mamma should be covered with a sinapism. It was allowed to remain on for thirty minutes; and on visiting her in six or seven hours after its removal, I found the whole right breast considerably swollen, hot, and painful. The next morning, the enlargement of the mamma was very much increased, the tumefaction having extended to the clavicle and axilla of the irritated side. There was no hard circumscribed or prominent tumour, but a painful, diffuse, elastic distension of the mammary gland, and surrounding cellular substance. On the evening of the day next succeeding the application of the sinapism, this poor girl with much joy reported that the catamenia had appeared. The flux having continued for two or three days in moderate quantity, she then found herself greatly relieved of the headache and other most distressing symptoms; and in a week her health was so far restored, that she ceased to require any farther attendance."

I am disposed to regard irritation of the mammæ as a convenient and rapid agency for the induction of menstruation; but one which must neither be rashly nor indiscriminately employed. In numerous cases it may be used alone; but, generally speaking, it may be advantageously combined with other means.

In cases of acute suppression of the menses, I am in the habit of prescribing, along with sinapisms to the mammæ, warm clothing of the bust and limbs, and the hot hip bath every twelve hours.

In anæmic amenorrhœa, it need hardly be stated, that irritation of the mammæ is only calculated to do good in conjunction with, or after a course of, a metallic medicine, such as some of the preparations of iron, manganese, or arsenic. In such cases, where we can trace a monthly ovarian nisus, though there be no catamenial flow, these periods should be seized as the appropriate times for using the sinapisms, and then also we may sometimes, by venturing a few doses of forcing medicine, such as cantharides and ergot, bring the case at once to a favorable issue.

The emmenagogue effects ascribed to the application of the leaves of the ricinus communis, by Drs. McWilliam and Tyler Smith, can easily be understood, when we remember their irritative character, and the consequences which we have found to be induced by irritation of the mammæ caused by other stimulants.

"When the breasts," says Dr. McWilliam, "are small and shrivelled, the plant is said to act more upon the uterine system, bringing on the menses if their period be distant, or causing their immoderate flow if their advent be near."

In the subjoined case, related by Dr. Tyler Smith, the effect produced may have been owing partly to the application to the breasts, and partly to the application to the genitals.

"I have used," says Dr. Tyler Smith, "the remedy in a case of scanty menstruation of a remarkable kind. Owing to exposure to marsh malaria some years ago, the patient had scarcely a sign of coloured discharge at the usual catamenial periods. She used the infusion of the leaves of the red bofareira at the date of her period, applying the infusion and leaves to the breasts, and the vapour to the genitals, with the effect of producing, in two days, a considerable flow of the catamenia."

III. *The leaves of the bofareira do not produce their Galactagogue and Emmenagogue Effects in virtue of any specific Property.*—The facts which have been already cited, point out pretty plainly that the effect of he leaves of the bofareira does not depend upon any specific property possessed by them; but simply on the determination produced by warmth and the irritative juices which they contain. That a good deal depends upon the mere warmth of the poultices, is sufficiently obvious. I have seen frequent examples in my own practice. I will not, however, enter into the particulars of these cases; but will conclude by mentioning two circumstances which thoroughly corroborate this view.

In the *Boletin de Medicine, Cirujia y Farmacia* of 14th November, 1852, a short abstract was given of Dr. McWilliam's paper. In the same journal of the 19th December following, a correspondent writes to say, that in consequence of the notice which had appeared of Dr. McWilliam's paper, he had used fomentations of fig-leaves *(hojas de higura)* to promote the secretion of milk in three cases, in which it had wholly or nearly ceased. In all the cases the benefit was decided. The Spanish practitioner wrote to confirm the practice recommended by Dr. McWilliam; but he has in reality, by using the wrong leaves, shown very clearly the galactagogue effects of poultices and fomentations, even when destitute of stimulating properties. I may likewise here state, upon the authority of a Spanish lady (with whom I conversed on this subject a few days ago), that in Cadiz, women are in the habit of bringing back the milk to their breasts after it has left them in consequence of weaning the child, or of any other cause, by means of drinking an infusion of the wild lupin *(altramuz)*, and applying to the mammæ fomentations made with the same plant. This plant has no stimulant or irritative quality, and the efficacy of the poultices made with it must, as in the case of the fig leaves, depend simply on heat and moisture, as in a common poultice. The internal use of the infusion might be dispensed with.—*American Journal Medical Sciences.*

